# Cast immobilization duration for distal radius fractures, a systematic review

**DOI:** 10.1007/s00068-024-02494-y

**Published:** 2024-03-20

**Authors:** Marcel A. N. de Bruijn, Laura A. van Ginkel, Emily Z. Boersma, Lysanne van Silfhout, Tjarda N. Tromp, Erik van de Krol, Michael J. R. Edwards, Vincent M. A. Stirler, Erik Hermans

**Affiliations:** 1 Department of Trauma Surgery, Radboud University Medical Center, Geert Grooteplein Zuid 10, Postbox 9101, 6500 HB Nijmegen, The Netherlands; 2 Faculty of Science and Technology - Technical Medicine, University of Twente, Enschede, The Netherlands; 3 Military Health Organisation, Ministry of Defense, Kromhout Kazerne, Utrecht, The Netherlands

**Keywords:** Distal radius fractures, Trauma, Conservative treatment, Duration of immobilization, Cast immobilization

## Abstract

**Purpose:**

The optimal duration of immobilization for the conservative treatment of non- or minimally displaced and displaced distal radius fractures remains under debate. This research aims to review studies of these treatments to add evidence regarding the optimal immobilization period.

**Methods:**

A comprehensive database search was conducted. Studies investigating and comparing short (< 3 weeks) versus long (> 3 weeks) immobilizations for the conservative treatment of distal radius fractures were included. The studies were evaluated for radiological and functional outcomes, including pain, grip strength, and range of motion. Two reviewers independently reviewed all studies and performed the data extraction.

**Results:**

The initial database search identified 11.981 studies, of which 16 (involving 1.118 patients) were ultimately included. Patient-reported outcome measurements, grip strength, range of motion, and radiological outcomes were often better after shorter immobilization treatments. Radiological outcomes were better with longer immobilization in two studies and shorter immobilization in one study. Fourteen studies concluded that early mobilization is preferred, while the remaining two studies observed better outcomes with longer immobilization. The data were unsuitable for meta-analysis due to their heterogeneous nature.

**Conclusion:**

Shorter immobilization for conservatively treated distal radius fractures often yield equal or better outcomes than longer immobilizations. The immobilization for non- or minimally displaced distal radius fractures could therefore be shortened to 3 weeks or less. Displaced and reduced distal radius fractures cannot be immobilized shorter than 4 weeks due to the risk of complications. Future research with homogeneous groups could elucidate the optimal duration of immobilization.

**Supplementary Information:**

The online version contains supplementary material available at 10.1007/s00068-024-02494-y.

## Background

Distal radius fractures (DRFs) are one of the most common fractures, and are often observed in young active patients and in patients aged 50 years and older. The overall incidence of DRFs is increasing due to the growing and aging population worldwide. Approximately 50% DRFs are treated conservatively [1–3]. However, there is no consensus about the optimal duration of immobilization for the conservative treatment of patients with DRFs.

In recent years, the operative treatment of patients with DRFs, including new minimally invasive surgical techniques, has been investigated [4]. Operative techniques to fixate a DRF include plating, minimally invasive percutaneous plate osteosynthesis, external fixation, and percutaneous pin fixation. There are several guidelines for the treatment and indications for the operative treatment of patients with DRFs [5, 6]. The American Academy of Orthopaedic Surgeons (AAOS) acknowledges that operative treatment leads to improved patient-reported and radiographic outcomes in patients with DRFs aged 65 years and younger, while in older patients, no difference was observed in patient-reported outcome measurements (PROMs) after 1 year whether they were treated operatively or conservatively. No recommendations or guidelines for the conservative treatment of patients with DRFs are provided by the AAOS, indicating the need for a unified treatment protocol [6].

To date, several systematic reviews have investigated the duration of immobilization for the conservative treatment of patients with DRFs. Although recommendations for the duration of immobilization are given, this did not result in a unified protocol. The review of the literature by van Delft et al. [7] included data from 12 articles and offers probably the most comprehensive analysis. These authors concluded that an immobilization period of 3 weeks or less is equally effective compared to longer immobilization, and might be associated with a better functional outcome [7]. Following the research by van Delft et al. [7], additional systematic reviews were performed by Cui et al. [8] and Østergaard et al. [9]. Cui et al. [8] focused their research on the safety of plaster splints compared with traditional small splints, such as wood chips, bamboo chips, or bark [10]. However, these authors also reported that plaster splints are more effective than traditional small splints when the intervention period is shortened (4 weeks compared with less than 4 weeks) [8]. Østergaard et al. [9] studied the benefits and harms of early mobilization after conservatively treating a patient with a DRF, reporting that no evidence supported the superiority of early or delayed mobilization, although the authors remark that longer immobilization may lead to physical inactivity [9].

According to the Dutch guideline for DRFs, primarily non-dislocated DRFs are immobilized for 3 weeks, and reduced DRFs are immobilized for 4 to 5 weeks [5]. There are several studies showing that a plaster cast treatment for a stable and non- or minimally displaced DRF for 1 week is safe and effective [7, 11, 12]. Furthermore, several studies conclude that immobilization of 1 to 3 weeks of plaster is preferred by patients. A feasibility trial from Boersma et al. [13] shows no significant difference in pain, functional outcome, or patient satisfaction between short and long immobilization in non-reduced DRFs, and no difference in occurrence of secondary displacement was found between the intervention and control groups [13].

The aim of this systematic review was to investigate the optimal duration of immobilization for the conservative treatment of DRFs in adults. To investigate whether the duration of immobilization can be safely shortened, special attention will be paid to articles that have not yet been featured in any systematic review regarding this topic. A distinction must be made for the duration of immobilization between the non-reduced and reduced DRF conservative treatments.

## Methods

### Protocol and registration

This systematic review was performed and reported according to the Preferred Reporting Items for Systematic Reviews and Meta-Analysis (PRISMA) guidelines [14]. The registration number in the International Prospective Registration of Systematic Reviews (PROSPERO) is CRD42023417924.

### Search strategy

A comprehensive literature search for studies comparing the duration of the conservative treatment of patients with DRFs was performed on January 24th, 2023. The searches were conducted using PubMed, Embase, CINAHL, Cochrane Library, and Web of Science. Index terms were determined for the literature search, and included Medical Subject Headings (MeSH) and closely related words. The following MeSH terms were used: “Radius Fractures,” “Wrist Fractures, “Conservative Treatment,” “Casts Surgical,” and “Splints.” No language or time restrictions were incorporated into the search. Other sources involved the manual screening of reference lists of randomized clinical trials, review articles, and systematic reviews. The Clinical Trial Register was checked for unpublished articles, and their authors were asked to give an update regarding their results. For a full search strategy, see Supplementary 1.

### Study selection

The studies retrieved from the searches were imported into Rayyan for Intelligent Systematic Review [15]. After dataset de-duplication, two reviewers (M.B. and L.G.) independently performed title and abstract screening to determine whether each study should be included in this review. Disagreements between the two authors were discussed, and a third reviewer (L.S.) was involved if consensus could not be reached.

### Eligibility criteria

All studies, randomized controlled trials (RCT) and otherwise, investigating the duration of conservative treatment for patients with DRFs were eligible for inclusion. The exclusion criteria consisted of (1) studies investigating the operative treatment of patients with DRFs, (2) pediatric patients (age < 18 years), (3) veterinarian studies, (4) the absence of the full text, and (5) case reports, editorials, conference abstracts, and letters to the editor. Studies in languages other than English, Dutch, or German were translated to review the abstracts for eligibility.

### Quality assessment

The included articles were assessed for their quality by two reviewers (M.B. and L.G.). The Cochrane Risk of Bias tool 2 (RoB2) was used to assess the risk of bias based on five domains: (1) randomization process, (2) deviations from intended interventions, (3) missing outcome data, (4) measurement of the outcome, and (5) selection of the reported result. The risk of bias was rated as low, some concerns, or high [16]. The Grading of Recommendations Assessment, Development and Evaluation (GRADE) was used to assess the quality of evidence, which was rated as very low, low, moderate, or high [17].

### Data extraction

Data extraction was performed independently by two reviewers with the use of a predefined data extraction form. The following characteristics were extracted from the included studies: author, year of publication, study design, number of included patients, follow-up period, non-operative immobilization treatment, reduction or none performed, duration of treatment, and outcome measurements (including wrist function, grip strength, range of motion, pain scores, and radiological outcome).

#### Analyzed outcome measurements from included articles

##### Patient-reported outcome measurements

The PROMs were evaluated with the Patient Rated Wrist Evaluation (PRWE); Disabilities of the Arm, Shoulder and Hand (DASH); Gartland and Werley; de Bruijn and the de Bruijn modified, Cooney, Patient-reported Outcome Measurement Information System Pain Interference (PROMIS-PI); and the Mayo Wrist score tools. The PRWE consists of three subscales: pain, function, and cosmetic. Participants are asked to respond to all items using a scale ranging from 0 to 10, with a total score of 100 [18]. The DASH and quick DASH (qDASH) tools assess the functional outcome of the upper extremity on a 5-point scale with 30- or 11-item questionnaires, respectively. Higher overall outcomes on the PRWE and (q-)DASH questionnaires represent a worse functional outcome [19]. The Gartland and Werly score combines subjective and objective items evaluating the wrist and hand function; here, a lower score represents a better functional outcome [20]. The de Bruijn and the modified de Bruijn scoring lists by Christersson et al. [21] evaluate functional outcome; again, a lower outcome represents a better wrist function [22]. The Cooney score assesses the domain’s pain, function, range of motion, and grip strength, with a total score of 100 points. In this score, a higher outcome over all domains represents a better functional outcome [21, 23]. The PROMIS-PI measures the extent to which pain limits a patient’s ability to engage in physical, mental, and social activities, with a lower overall outcome representing less pain interference [24]. Finally, the Mayo Wrist Score represents pain during the active motion of the injured wrist compared to the contralateral wrist and indicates the possibility of resuming daily activities; it is measured on a scale from 0 to 100, where a higher overall outcome represents a better wrist function [25].

##### Pain scores

Pain scores were either measured using a visual analog scale (VAS) or as a percentage of normal and mild pain.

##### Grip strength

Grip strength was measured using hand-grip dynamometry. It was expressed as mean grip strength of the injured limb and grip strength of the injured limb as a percentage of the contralateral wrist.

##### Range of motion

Range of motion was measured by joint extension, flexion, and deviation. It was expressed as degrees of motion, forearm rotation, mean range of motion, mean deviations of the injured wrist expressed as percentages of the uninjured wrist, and as the sum of flexion, extension, and radial and ulnar deviation.

##### Radiological outcome

Radiological outcome was measured using the Lidström criteria. The results were presented as excellent, good, fair, or poor based on anatomical outcomes expressed as percentages [26]. Measurements of radial and volar angulation in degrees, radial length, and shortening in millimeters were used to determine anatomical differences during treatment.

### Statistical analysis

The data were collected and analyzed using the Review Manager software (version 5.3). The protocol and study population were investigated to determine clinical homogeneity. Statistical homogeneity was determined by the use of *I*^2^ tests, with values less than 40% considered homogeneous [27]. Funnel plots were generated using Review Manager to determine the publication bias [28].

## Results

### Study selection

**Fig. 1:**
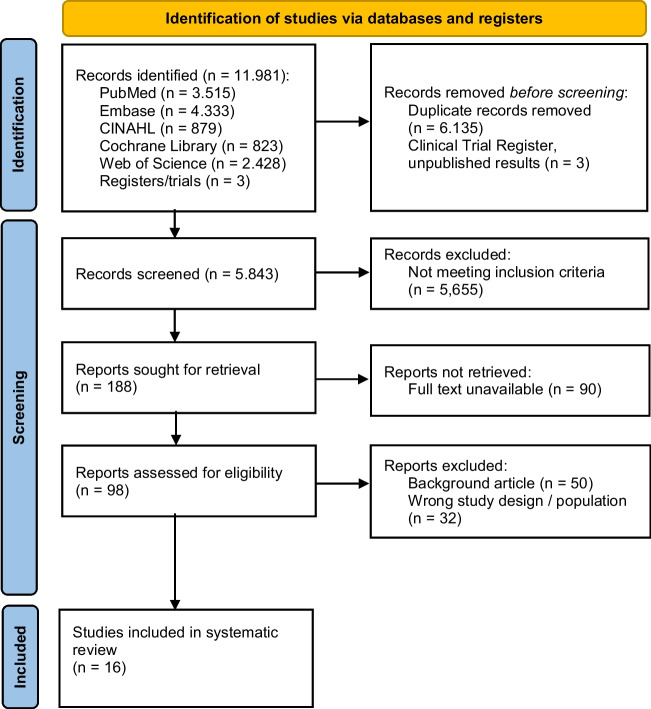
Article selection based on the Preferred Reporting Items for Systematic Reviews and Meta-Analyses guidelines (PRISMA)

The initial literature search resulted in 11.981 articles. After de-duplication, 5.843 articles were screened for title and abstract, leaving 188 articles to be screened as full texts; however, the full texts of 90 studies were unavailable. Background articles, encompassing a total of 50 studies, which reviewed current treatment approaches, emerging trends in treatment management, or provided background information for DRFs were excluded. A further 32 studies were excluded because they used the wrong study design or study population for this review, mainly focusing on comparisons between the duration of immobilization of surgical treatment and nonsurgical treatment. A total of 16 articles were included in this systematic review (see Fig. 1).

### Study characteristics

**Fig. 2:**
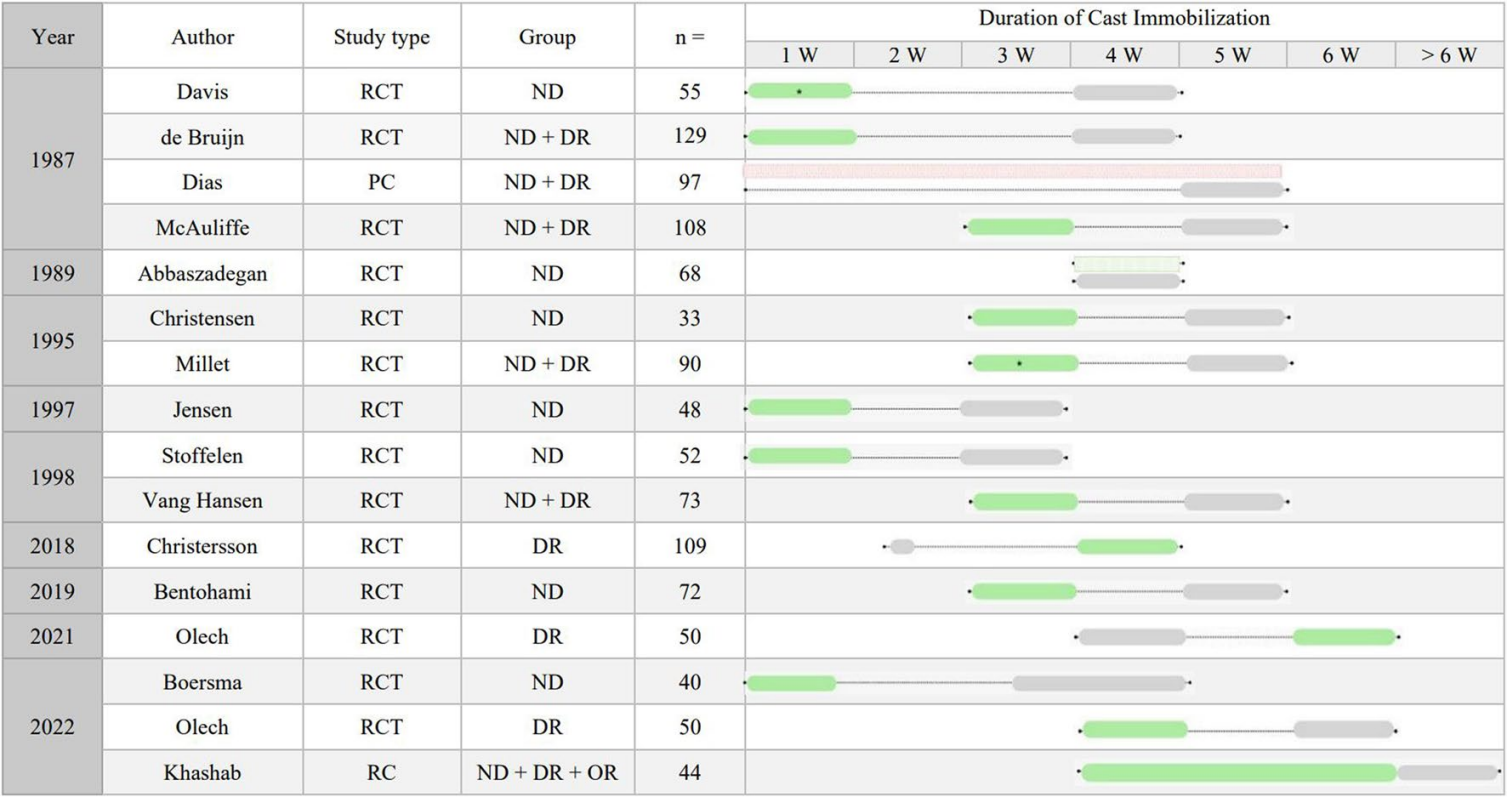
Overview of the included articles representing the investigated duration of immobilization within each study. The outer limits of the immobilization durations are marked with black dots. The preferred duration of immobilization according to the authors’ conclusions is marked green. Gray boxes represent unfavorable duration of immobilization. The use of only bandage as a treatment is marked as a striped rounded box. An unknown immobilization duration is presented in red. Abbreviations: ND non- or minimally displaced, DR displaced and reduced, OR operation, RC retrospective cohort, W weeks, * = additional immobilization is given after the removal of the cast

In total, 1118 patients were included (male/female/unknown gender: 205/860/53). All of the included studies investigated the duration of immobilization of patients with DRFs. One study compared bandages, with an unknown duration of immobilization, with cast immobilization for 5 weeks [29]. Another study retrospectively investigated immobilization of less or more than 6 weeks and was therefore included [30]. All other studies compared the differences between two groups, both of which were treated with less than 6 weeks of immobilization. Groups were divided into either shorter immobilization (< 3 weeks) or longer immobilization (> 3 weeks) periods. An overview of the selected articles, study characteristics, and preferred duration of immobilization concluded by the author is provided in Fig. 2.

### Patient-reported outcome measurements—function

PROMs were reported in 10 studies [11, 13, 21, 22, 29, 31–35]. Four studies showed significantly better functional outcomes for the shorter rather than longer immobilization group in terms of the Gartland and Werley score, PRWE, qDASH, and PROMIS PI [13, 22, 31, 32]. Only one study showed that the longer immobilization group had a significantly better outcome in the Gartland and Werley score at the time of cast removal [11]. All other studies showed no significant differences (see Table 1).

**Table 1:** Overview of patient-reported outcome measurements. Abbreviations: *ND* non- or minimally displaced, *DR* displaced and reduced, *SD* standard deviation, *W* weeks, *M* months, *Y* years, *PRWE* Patient Rated Wrist Evaluation, (*q-)DASH* (quick) Disabilities of the Arm, Shoulder and Hand, *PROMIS-PI* patient-reported Outcomes Measurement Information System (PROMIS) Pain Interference

Wrist function								
Author	Fracture type	Follow up after trauma	Outcome measurement				*P*-value	Outcome measurement type
			Short immobilization	SD	Long immobilization	SD		
Davis [32] (1987)	ND	5 W	29.63	-	6.25	-	*P* < 0.05	Gartland and Werly’s (% excellent score)
		7 W	44.44	-	4.00	-	*P* = 0.01	Gartland and Werly’s (% excellent score)
de Bruijn [22] (1987)	ND + DR	4 W	36.52	24.51	49.14	27.18	*P* = 0.01	de Bruijn
		6 W	25.74	20.14	33.24	23.73	*P* = 0.11	de Bruijn
		10 W	15.72	12.64	20.82	18.66	*P* = 0.28	de Bruijn
		14 W	13.82	9.70	15.52	12.51	*P* = 0.78	de Bruijn
		26 W	7.83	5.76	10.01	9.91	*P* = 0.53	de Bruijn
		52 W	4.56	4.38	6.33	5.92	*P* = 0.20	de Bruijn
Dias [29] (1987)	ND	13 W	24	-	6.4	-	-	Gartland and Werly’s (% excellent score)
	DR	13 W	16.3	-	6.4	-	-	Gartland and Werly’s (% excellent score)
McAuliffe [33] (1987)	DR	12 W	72	-	66	-	-	Gartland and Werly’s (% excellent score and good)
		1 Y	85	-	77	-	-	Gartland and Werly’s (% excellent score and good)
Jensen [11] (1997)	ND	1 W	9.1	-	15.4	-	*P* = 0.05	Gartland and Werly’s (% excellent score)
		3 M	18.2	-	38.5	-	*P* < 0.3	Gartland and Werly’s (% excellent score)
		26 W	68.2	-	88.5		*P* < 0.4	Gartland and Werly’s (% excellent score)
Stoffelen [35] (1998)	ND	6 W	61.6	12.1	56.8	19.7	*P* = 0.29	Cooney score
		3 M	77.4	13.8	71.5	19.2	*P* = 0.19	Cooney score
		6 M	84.6	11.6	81.3	19.33	*P* = 0.45	Cooney score
		1 Y	86.8	10.9	82.2	18.6	*P* = 0.27	Cooney score
Christersson [21] (2018)	DR	1 Y	4.8	3.4	4.3	3.0	*P* = 0.38	Gartland and Werley’s (0–35)
			22.7	14.3	22.9	14.9	*P* = 0.96	de Bruijn modified (0–154)
			74.4	11.3	73.6	12.8	*P* = 0.73	Mayo-modified (0–100)
Bentohami [31] (2018)	ND	6 W	20	-	30.7	-	*P* = 0.32	PRWE
			13.6	-	22.4		*P* = 0.74	qDASH
		12 W	10	-	24.3	-	*P* = 0.05	PRWE
			14.7	-	20.5	-	*P* = 0.34	qDASH
		6 M	9.5	-	8.3	-	*P* = 0.33	PRWE
			4.5	-	4.5	-	*P* = 0.95	qDASH
		1 Y	5.0	-	8.8	-	*P* = 0.05	PRWE
			0.0	-	12.5	-	*P* = 0.03	qDASH
Boersma [13] (2022)	ND	6 W	24.5	22.1	33.1	18.5	*P* = 0.25	PRWE
			15.6	15.5	28.8	16.6	*P* = 0.05	DASH
			51.6	6.4	56.7	6.8	*P* = 0.04	PROMIS-PI
		3 M	10.2	11.4	15.8	15.3	*P* = 0.29	PRWE
			9.3	10.6	10.6	6.7	*P* = 0.76	DASH
			44.2	6.7	49.9	7.0	*P* = 0.03	PROMIS-PI
		6 M	7.2	12.0	10.9	12.7	*P* = 0.44	PRWE
			5.7	8.6	6.8	5.7	*P* = 0.73	DASH
			44.4	5.6	45.8	5.7	*P* = 0.49	PROMIS-PI
		1 Y	2.9	7.6	2.3	3.1	*P* = 0.82	PRWE
			1.6	2.5	6.6	10.3	*P* = 0.12	DASH
			41.5	2.7	47.0	7.8	*P* = 0.03	PROMIS-PI
Olech [34] (2022)	DR	1 Y	58.46	21.24	61.87	22.97	*P* = 0.59	Mayo wrist score

### Pain

Pain was described in ten studies, mostly expressed as VAS or as a percentage of normal and mild pain [13, 21, 29–34, 36, 37]. Two studies showed a significantly better outcome in the shorter immobilization group [33, 36]. Although not significantly different, most studies with a short immobilization duration showed lower pain scores, except in one study for which a better outcome was reported in the longer immobilization group (see Table 2) [21].

**Table 2:** Overview of studies investigating pain, measured on the visual analog scale (VAS) or as a percentage of no or mild pain. + / + outcome favorable in this group and -/- outcome unfavorable in this group. Abbreviations: *ND* non- or minimally displaced, *DR* displaced and reduced, *OR* operated, *SD* standard deviation, *W* weeks, *M* months, *Y* years

Pain								
Author	Fracture type	Follow up after trauma	Outcome measurement				*P*-value	Outcome measurement type
			Short immobilization	SD	Long immobilization	SD		
Davis [32] (1987)	ND	1 W	6.4	6.2	8.3	4.9	-	VAS (0–20)
		4 W	4.0	4.4	5.8	4.7	-	VAS (0–20)
		6 W	2.6	3.1	3.6	3.2	-	VAS (0–20)
Dias [29] (1987)	ND	0 W	57	-	54	-	-	VAS (0–100)
		1 W	26	-	30	-	-	VAS (0–100)
		5 W	13	-	22	-	-	VAS (0–100)
		9 W	9	-	17	-	-	VAS (0–100)
		13 W	9	-	14	-	-	VAS (0–100)
	DR	0 W	68	-	68	-	-	VAS (0–100)
		1 W	3	-	38	-	-	VAS (0–100)
		5 W	14	-	19	-	-	VAS (0–100)
		9 W	13	-	18	-	-	VAS (0–100)
		13 W	13	-	22	-	-	VAS (0–100)
McAuliffe [33] (1987)	ND + DR	0 W	+ / +	-	- / -	-	*P* = 0.004	VAS (0–10)
		3 M	+ / +	-	- / -	-	*P* = 0.056	VAS (0–10)
		1 Y	+ / +	-	- / -	-	*P* = 0.02	VAS (0–10)
Abbaszadegan [36] (1987)	ND	11 D	4.0	-	4.7		*P* = 0.09	VAS (0–10)
		4 W	3.4	-	3.7	-	*P* = 0.4	VAS (0–10)
		8 W	1.8	-	3.2	-	*P* < 0.001	VAS (0–10)
		1 Y	1.3	-	1.9		*P* = 0.06	VAS (0–10)
Vang Hansen [37] (1998)	ND + DR	1 Y	76	-	78	-	-	Pain with strenuous use (% no versus mild pain)
Christersson [21] (2018)	DR	10 D	2.5	-	2.0		*P* = 0.41	VAS (0–10)
		1 M	1.6	-	1.0	-	*P* = 0.06	VAS (0–10)
		4 M	0.5	-	0.4	-	*P* = 0.67	VAS (0–10)
		1 Y	0.2	-	0.1	-	*P* = 0.92	VAS (0–10)
Bentohami [31] (2019)	ND	3 W / 5 W	3.1	-	2.6	-	*P* = 0.46	VAS (0–10)
Boersma [13] (2022)	ND	4 W	2	-	2.2	-	*P* = 0.73	VAS (0–10)
Olech [34] (2022)	DR	1 Y	2.53	3.06	3.58	2.56	*P* = 0.199	VAS (0–10)
			7.61	1.83	7.58	2.3	*P* = 0.957	Activity score VAS (0–10)
Khashab [30] (2022)	ND + DR + OR	1Y	5.6	-	16.7	-	-	Pain score (% mild pain)

### Grip strength

Grip strength was described in nine studies [21, 29, 30, 32, 33, 36–39]. Four studies showed a significantly better outcome in the shorter immobilization group [29, 33, 36, 38]. Only one study reported a significantly better grip strength in the group with a longer (6 weeks) immobilization [39]. All other studies showed no significant differences (see Table 3).

**Table 3:** Overview of studies investigating grip strength. Expressed as the mean grip strength of the injured limb and grip strength of the injured limb as a percentage of the contralateral wrist. + / + outcome favorable in this group and -/- outcome unfavorable in this group. Abbreviations: *ND* non- or minimally displaced, *DR* displaced and reduced, *OR* operated, *RC* retrospective cohort, *SD* standard deviation, *W* weeks, *M* months, *Y* years

Grip strength								
Author	Fracture type	Follow up after trauma	Outcome measurement				*P*-value	Outcome measurement type
			Short immobilization	SD	Long immobilization	SD		
Davis [32] (1987)	ND	2 W	73	24	70	34	-	Mean grip strength of the injured side (mmHg)
		5 W	147	62	115	62	-	Mean grip strength of the injured side (mmHg)
		7 W	162	63	138	64	-	Mean grip strength of the injured side (mmHg)
Dias [29] (1987)	ND	5 W	57.1	-	36.1	-	*P* = 0.000	Grip strength recovery (% of the strength of the contralateral hand)
		9 W	63.5	-	45.7	-	*P* = 0.005	Grip strength recovery (% of the strength of the contralateral hand)
		13 W	76.2	-	58.3	-	*P* = 0.000	Grip strength recovery (% of the strength of the contralateral hand)
	DR	5 W	33.4	-	25.0	-	*P* = 0.016	Grip strength recovery (% of the strength of the contralateral hand)
		9 W	48.8	-	44.0	-	*P* = 0.215	Grip strength recovery (% of the strength of the contralateral hand)
		13 W	62.7	-	60.1	-	*P* = 0.540	Grip strength recovery (% of the strength of the contralateral hand)
McAuliffe [33] (1987)	ND + DR	1 Y	+ / +	-	- / -	-	*P* = 0.001	Grip strength (unit not specified)
Abbaszadegan [36] (1987)		4 W	52	-	40		*P* = 0.06	Grip strength recovery (% of the strength of the contralateral hand)
	ND	8 W	67	-	60	-	*P* = 0.25	Grip strength recovery (% of the strength of the contralateral hand)
		1 Y	94	-	78	-	*P* = 0.045	Grip strength recovery (% of the strength of the contralateral hand)
Vang Hansen [37] (1998)	ND + DR	1 Y	83	-	90	-	-	Grip strength recovery (% of the strength of the contralateral hand)
Christersson [21] (2018)		1 M	− 17.4	-	− 19.6	-	*P* = 0.14	Grip strength recovery (% of the strength of the contralateral hand)
	DR	4 M	− 9.6	-	− 9.6	-	*P* = 0.94	Grip strength recovery (% of the strength of the contralateral hand)
		1 Y	− 4.4	-	− 4.4	-	*P* = 0.94	Grip strength recovery (% of the strength of the contralateral hand)
Olech [39] (2021)	DR	1 Y	71.12	14.24	81.07	12.59	*P* = 0.032	Grip strength recovery (% of the strength of the contralateral hand)
			25.45	12.53	27.6	11.46	*P* = 0.532	Grip power treated limb (kg)
Khashab [30] (2022)	ND + DR + OR	RC	7.14	-	25	-	*P* = 0.291	Grip rate (% of normal)

### Range of motion

Range of motion was described in eight studies [21, 29, 30, 33, 36–39]. Four studies showed a significantly better outcome in the shorter immobilization group [21, 33, 36, 38]. One study reported a better range of motion when given prolonged immobilization [39]. No significant differences were shown in the other studies (see Table 4).

**Table 4:** Overview of studies investigating range of motion, measured by joint extension, flexion, and deviation. Expressed as (1) degrees of motion, (2) forearm rotation, (3) mean range of motion, (4) mean deviations of the injured wrist expressed as percentages of the uninjured wrist, and (5) as the sum of flexion, extension, and radial and ulnar deviation. Abbreviations: *ND* non- or minimally displaced, *DR* displaced and reduced, *SD* standard deviation, *W* weeks, *M* months, *Y* years

Range of motion								
Author	Fracture type	Follow up after trauma	Outcome measurement				*P*-value	Outcome measurement type
			Short immobilization	SD	Long immobilization	SD		
Dias [29] (1987)	ND	5 W	76	-	41	-	-	Sum of flexion, extension, radial deviation, and ulnar deviation (% of contralateral side)
		9 W	85	-	61	-	-	Sum of flexion, extension, radial deviation, and ulnar deviation (% of contralateral side)
		13 W	91	-	74	-	-	Sum of flexion, extension, radial deviation, and ulnar deviation (% of contralateral side)
	DR	5 W	54	-	28	-	-	Sum of flexion, extension, radial deviation, and ulnar deviation (% of contralateral side)
		9 W	74	-	54	-	-	Sum of flexion, extension, radial deviation, and ulnar deviation (% of contralateral side)
		13 W	83	-	74	-	-	Sum of flexion, extension, radial deviation, and ulnar deviation (% of contralateral side)
McAuliffe [33] (1987)	ND + DR	3 M	24	-	21	-	-	Palmairflexion (degrees)
			47	-	46	-	-	Dorsiflexion (degrees)
			34	-	32	-	-	Radial deviation (degrees)
			25	-	21	-	-	Uinar deviation (degrees)
			83	-	77	-	-	Pronation (degrees)
			75	-	71	-	-	Supination (degrees)
		1 Y	27	-	24	-	-	Palmairflexion (degrees)
			57	-	54	-	-	Dorsiflexion (degrees)
			37	-	39	-	-	Radial deviation (degrees)
			25	-	23	-	-	Ulnar deviation (degrees)
			92	-	81	-	*P* = 0.02	Pronation (degrees)
			82	-	73	-	-	Supination (degrees)
Abbaszadegan [36] (1987)	ND	4 W	80	-	58	-	*P* < 0.001	Extension + flexion (mean values in % of the uninjured wrist)
		8 W	90	-	75	-	*P* < 0.001	Extension + flexion (mean values in % of the uninjured wrist)
			86	-	66	-	*P* < 0.001	Radial + ulnar deviation (mean values in % of the uninjured wrist)
		1 Y	98	-	89	-	*P* = 0.002	Extension + flexion (mean values in % of the uninjured wrist)
			98	-	90	-	*P* = 0.007	Radial + ulnar deviation (mean values in % of the uninjured wrist)
Millet [38] (1995)	ND + DR	5 W	169.6	80.6	143.4	80.6	-	Mean range of motion (sum of active, painless flexion, extension, pronation, and supination)
		3 M	280.1	59.7	252.1	72.1	*P* < 0.05	Mean range of motion (sum of active, painless flexion, extension, pronation, and supination)
		6 M	303.6	54.3	290.6	55.8	-	Mean range of motion (sum of active, painless flexion, extension, pronation, and supination)
		3 Y	317.1	21.8	304.7	49.7	-	Mean range of motion (sum of active, painless flexion, extension, pronation, and supination)
Vang Hansen [37] (1998)	ND + DR	1 Y	130	-	120	-	-	Range of forearm rotation (degrees)
Christersson [21] (2018)	DR	1 M	–22.5	-	–36.25	-	*P* < 0.001	Dorsal extension (in degrees, compared with uninjured side)
			–33.75	-	–31.25	-	*P* = 0.38	Volar flexion (in degrees, compared with uninjured side)
			–13.75	-	–25	-	*P* = 0.003	Pronation (in degrees, compared with uninjured side)
		4 M	–7.5	-	–6.25	-	*P* = 0.59	Dorsal extension (in degrees, compared with uninjured side)
			–21.25	-	–18.75	-	*P* = 0.39	Volar flexion (in degrees, compared with uninjured side)
			–3.75	-	–6.25	-	*P* = 0.21	Pronation (in degrees, compared with uninjured side)
		1 Y	–0.625	-	1.857	-	*P* = 0.43	Dorsal extension (in degrees, compared with uninjured side)
			–13.75	-	–8.75	-	*P* = 0.13	Volar flexion (in degrees, compared with uninjured side)
			–8.13	-	–6.25	-	*P* = 0.39	Pronation (in degrees, compared with uninjured side)
Olech [39] (2021)	DR	1 Y	61.53	9.1	74.87	10.66	*P* = 0.025	Flexion treated limb (degrees)
			50.17	17.47	57.02	17.34	*P* = 0.171	Extension treated limb (degrees)
			33.25	13.22	39.54	15.41	*P* = 0.127	Uinar deviation treated limb (degrees)
			18.59	11.7	21.18	15.31	*P* = 0.503	Radial deviation treated limb (degrees)

### Radiological outcome

Radiological outcome was described in 10 studies [11, 22, 29, 31, 33, 34, 36–38, 40]. Three studies reported a significantly better outcome for patients treated with a longer immobilization for displaced and reduced DRFs [22, 34, 38]. Only one study showed a significantly better outcome on the Lidström criteria if a shorter immobilization was performed [36]. All other studies showed no significant differences (see Table 5).

**Table 5:** Overview of studies investigating radiological outcome. Presented as a percentage of excellent scores on the Lidström criteria or measured as radial and volar angulation in degrees, radial length, and shortening in millimeters. Abbreviations: *ND* non- or minimally displaced, *DR* displaced and reduced, *SD* standard deviation, *W* weeks, *M* months, *Y* years

Radiological outcome								
Author	Fracture type	Follow up after trauma	Outcome measurement				*P*-value	Outcome measurement type
			Short immobilization	SD	Long immobilization	SD		
de Bruijn [22] (1987)	ND	1 Y	7.36	-	6.89	-	*P* = 0.62	Volar angle difference
			2.55	-	1.50	-	*P* = 0.79	Radial angle difference
			1.91	-	0.73	-	*P* = 0.30	Radial length difference
			1.55	-	1.31	-	*P* = 0.88	Radial shift
	DR	1 Y	13.59	-	13.65	-	*P* = 0.38	Volar angle difference
			8.09	-	5.02	-	*P* = 0.02	Radial angle difference
			5.56	-	3.22	-	*P* = 0.01	Radial length difference
			3.56	-	2.67	-	*P* = 0.32	Radial shift
Dias [29] (1987)	ND	13 W	22.0	-	4.3	-	-	Lidström anatomical outcome (% excellent)
	DR		8.5	-	7.0	-	-	Lidström anatomical outcome (% excellent)
McAuliffe [33] (1987)	ND + DR	3 M	8.5	-	8.9	-	-	Dorsal angulation (degrees)
			4.2	-	4.8	-	-	Radial angulation (degrees)
			3.3	-	3.8	-	-	Radial shortening (mm)
Abbaszadegan [36] (1989)	ND	1 Y	91.2	-	67.6	-	*P* < 0.05	Lidström grading (% excellent)
Christensen [40] (1995)	ND	9 M	3.1	-	4.4	-	-	Dorsal angulation (degrees)
			1.9	-	2.0	-	-	Radial angulation (degrees)
			1.5	-	1.2	-	-	Axial radial length change (mm)
Millet [38] (1995)	ND + DR	3 Y	4.6	-	6.2	-	-	Dorsal angulation (degrees)
			2.5	-	0.6	-	*P* < 0.05	Radial angulation (degrees)
			2.8	-	3.4	-	-	Radial shortening (mm)
Jensen [11] (1997)	ND	26 W	100	-	88.5	-	-	No angulation (degrees)
			81.8	-	73.1	-	-	No axial radial shortening (% per group)
			13.6	-	23.1	-	-	Shortening of 1–2 mm (% per group)
			4.5	-	3.8	-	-	Shortening of 23 mm (% per group)
Vang Hansen [37] (1997)	ND + DR	1 Y	4	-	5	-	-	Dorsal angulation (degrees)
			9	-	9	-	-	Radial length difference (mm)
Bentohami [31] (2019)	ND	1 Y	97	-	97	-	-	Lidström grading (% excellent)
Olech [39] (2021)	DR	1 Y	1.9	1.62	2.45	2.47	*P* = 0.35	Radial height (degrees)
			9.13	7.12	3.29	5.11	*P* = 0.04	Volar tit (degrees)
			0.55	2.84	0.25	1.03	*P* = 0.62	Radial height (mm)

### Risk of bias

**Fig. 3:**
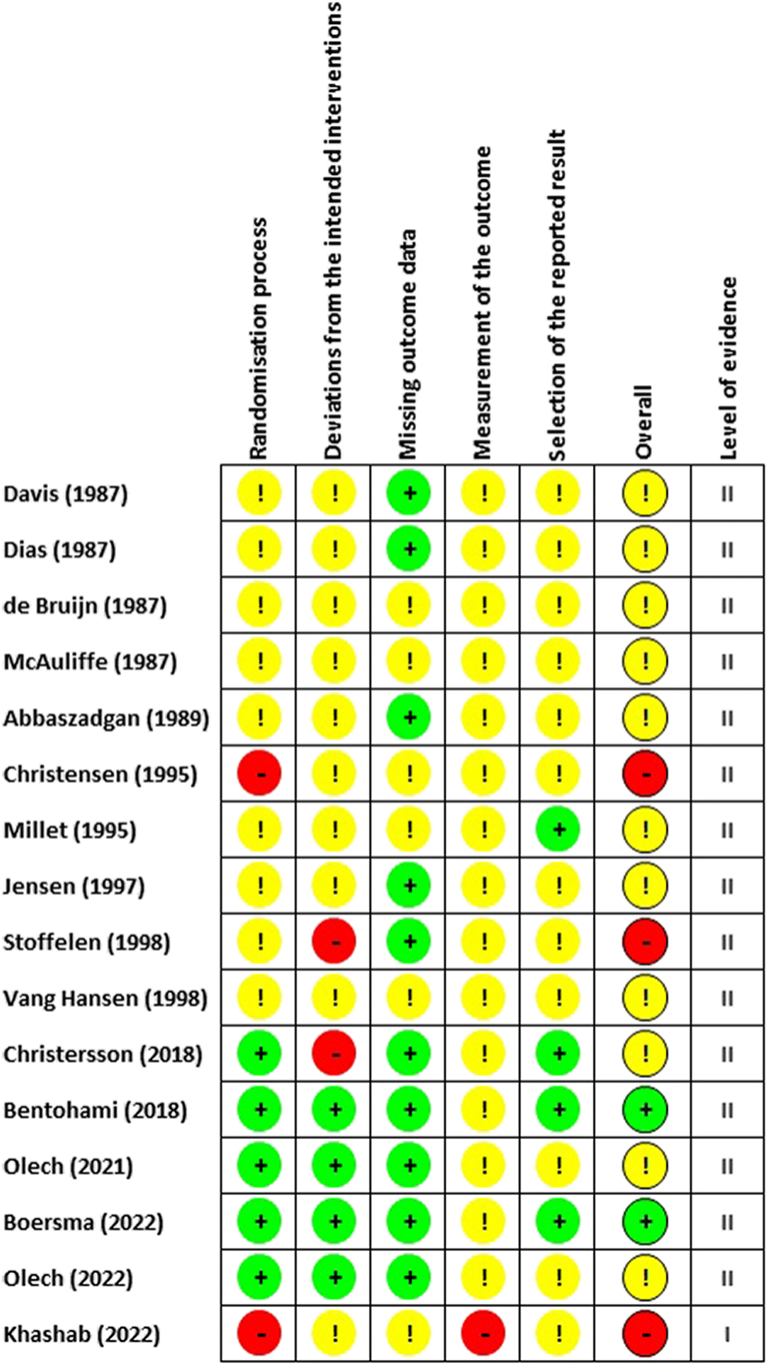
Risk of bias assessment and level of evidence

All studies were assessed with the Cochrane Risk of Bias Tool 2 (RoB2). Two studies were assessed as low risk [13, 31], while 11 studies were assessed to present some concerns [7, 21, 22, 29, 32–34, 36–39]. Three studies were considered to have a high risk of bias [30, 35, 40] (see Fig. 3).

### Level of evidence

All studies were assessed with GRADEpro to qualify the level of evidence [17]. A total of 15 studies were qualified as level II evidence [7, 13, 21, 22, 29, 31–40], and one study was qualified as level I evidence [30] (see Fig. 3).

## Discussion

This systematic review summarized and analyzed the optimal duration of immobilization for the conservative treatment of patients with DRFs. The main finding to emerge from the analysis is that 14 of the 16 included studies reported the possible benefit of a shorter immobilization period. The authors concluded that shorter immobilization resulted in the early recovery of range of motion and improved grip strength without increasing discomfort, but did not worsen anatomical outcome and were clinically equivalent to longer immobilization. The recovery of patients with a non- or minimally displaced fracture was favorable when treated with 3 weeks or less of immobilization [11, 13, 22, 29, 32–38, 40]. The immobilization of displaced and reduced DRFs can be shortened a minimum of 4 weeks. Two studies did not support a shorter immobilization due to treatment failure, reporting better outcomes in their longer immobilization groups, both of which included displaced and reduced DRFs. One study investigated immobilizations of 10 days and 4 weeks, while the other study compared immobilizations of 4 and 6 weeks [21, 39].

The last comprehensive systematic review was performed in 2018 by van Delft et al. [7] and concluded that the period of immobilization for all DRFs should be considered to be shortened to a maximum of 3 weeks [7]. Since 2018, another four studies investigated the duration of immobilization for conservatively treated DRFs [13, 30, 34, 39]. Functional outcome, pain, range of motion, radiological outcomes, and grip strength were the most commonly used outcome measurements. Across all the included studies in this review, these outcome measurements differ in terms of outcome values. Standard deviations and interquartile ranges were often not reported. In addition, studies pooled outcome values for the non- and minimally displaced and displaced DRFs [22, 29, 30, 33, 37, 38]. The finding of this systematic review confirms the conclusion of the last systematic review performed, that there is a lack of clear homogeneous studies and a need for these studies in future research. The present systematic review included four new studies, of which three showed beneficial outcomes using shorter immobilization. Three of the recent studies also had a better risk of bias than the previous studies. This shows that the overall consensus from recent studies leans more towards the use of shorter immobilization for the conservative treatment of patients with DRFs. Due to the heterogeneity of study designs, a meta-analysis could not be performed.

Christersson et al. [21] investigated whether 10 days of immobilization was sufficient for the treatment of patients with reduced DRFs. No significant difference was observed in pain scores or grip strength, but there was a significantly better outcome in range of motion for the longer immobilization group. Furthermore, the treatment with shorter immobilization failed in three patients. One patient received a cast immobilization for another 3 weeks, while two patients were treated surgically due to secondary dislocation. It was concluded that 10 days of immobilization after the reduction of a DRF is not safe and causes more complications [21]. One must be aware that only displaced DRFs that needed fracture reduction were included in this study. This contributes to previous findings that displaced and reduced DRFs cannot be immobilized safely for less than 4 weeks. Distinctions should therefore be made between the non- and minimally displaced fractures in comparison with displaced fractures for the conservative treatment of patients with DRFs.

Olech et al. [34, 39] investigated the duration of immobilization used to treat displaced and reduced DRFs in the elderly population in two studies. The first study (2021) showed a better recovery of muscle strength and range of motion in the group that underwent 6 weeks compared to 4 weeks of immobilization, and the authors concluded that a more intensive rehabilitation process is preferred [39]. The second study (2022) of the same study population explored pain scores and the Mayo wrist function score after 4 weeks of immobilization, concluding that immobilization can be reduced to 4 weeks for conservative DRF treatment [34]. As mentioned by the AAOS, operative treatments of DRFs in the elderly do not lead to functional differences in PROMs compared with conservative treatments [6]. When better functional outcomes can be obtained with shorter immobilization in the elderly, as concluded by Olech et al. (2022), one must consider this the optimal treatment for this specific group of patients.

Boersma et al. [41] investigated 1 week of immobilization in comparison with 4 to 5 weeks, with a total of 40 patients. They found no significant differences in pain between the two groups, while the shorter immobilization led to better functional outcomes after 6 weeks and an overall better patient satisfaction [13]. This feasibility study led to the Cast-OFF 2 study, in which a multicenter randomized controlled trial with a stepped wedge design was used to implement one week of immobilization for the non- or minimally displaced DRFs. This study should provide more evidence from a homogeneous population [41].

One retrospective study by Khashab et al. [30] was included as they investigated the duration of immobilization during the DRF treatments. Non- or minimally displaced, displaced and reduced, or surgically treated DRFs were included and pooled, but no distinction was made between the conservative and surgically treated DRFs. According to Khashab, decreased grip strength and higher pain scores were observed if the immobilization exceeded more than 6 weeks. The duration of prolonged immobilization and the kind of treatment the patients received was unclear however [30]. Furthermore, it should be noted that, due to the retrospective design of this study, the risk of bias was assessed as high, based on the domain of randomization and measurement of the outcome. The RoB2 tool was designed for randomized trials, and this study shows limitations in assessing retrospective studies for this systematic review.

The benefits of early mobilization were not only observed after the conservative treatment of DRFs; rather, this was also concluded after surgical DRF treatment in a systematic review performed by Deng et al. [42]. Nine randomized control trials were investigated to explore the differences between early and late mobilization, respectively after less than 2 weeks or more than 2 weeks of immobilization following the surgical treatment of patients with DRFs. Early mobilization showed better functional outcomes at earlier post-operative stages and similar clinical outcomes during long-term follow-up; however, it must be noted that early mobilization had a higher potential for osteosynthesis failure. It was concluded that early mobilization could be considered, although more research must be performed investigating the optimal rehabilitation protocol for surgically treated DRFs [42].

This systematic review had a few limitations. First, a comprehensive literature search was performed without restriction to screen all eligible studies. Potential language barriers, other than English, Dutch, or German, were translated to screen for eligibility. This could have led to missed inclusions, despite the article selection being performed by two individual reviewers. The full text was not available for a large proportion (48%) of the eligible articles, even after the help and in-depth searching of a librarian. This could have led to potential missed inclusions. Second, only 4 new eligible articles were included since the systematic review performed by van Delft et al. [7], of which 3 of these articles, assessed to have a lower risk of bias, provided more evidence in favor of shorter immobilizations. It was aimed to include more articles with homogeneous studies to perform a meta-analysis. Although these 4 articles mostly did report standard deviations or interquartile ranges, different outcome measurements were used [13, 30, 34, 39]. In combination with the heterogeneous studies from most of the older included articles, which were 20 years old or more, it was not possible to perform a meta-analysis. Third, it must be noted that not all authors conducted a minimal follow-up of 1 year; several studies had a shorter follow-up ranging from 7 weeks to 9 months [11, 29, 32, 40]. There is no established standard for fracture follow-up in clinical research, although a 1-year period is frequently utilized and recommended [43]. Assessments of outcomes at week 6, month 3, month 6, month 9, and month 12 can be crucial for evaluating complications, pain, and functionality. Studies with a follow-up duration of less than 1 year may yield short-term benefits, but may be unfavorable when evaluating long-term outcomes.

The results of this study indicate that the duration of immobilization for the conservative treatment of patients with DRFs is not widely investigated. Of the 16 studies included, ten were published more than 20 years ago. It was not until 2018 that several researchers started to reinvestigate this topic. Future research must be performed with homogeneous studies and outcome measurements to investigate the optimal duration of immobilization. Distinctions should be made between the conservative treatment of the non- or minimally displaced DRFs, and the displaced DRFs. Several studies have investigated this topic, although without published results yet [41, 44–46]. Hopefully, these and future studies will lead to a uniform protocol for the optimal duration of immobilization during the conservative treatment of DRFs.

## Conclusion

This comprehensive systematic review showed the same or better outcome measurements of shorter immobilization for the non- or minimally displaced DRFs. The duration of immobilization for non- or minimally displaced DRFs can be considered to be shortened to 1 to 3 weeks. Displaced and reduced DRFs cannot be immobilized for less than 4 weeks as this results in more complications. Future large homogeneous randomized controlled trials should provide definitive evidence to reach a consensus on the optimal duration of immobilization.

## Supplementary Information

Below is the link to the electronic supplementary material.

Supplementary file1 (DOCX 20 KB)

## Data Availability

The data utilized for this systematic review is available upon reasonable request.

## Declarations

### Systematic review registration number

Prospero CRD42023417924.
