# Supplementary material for: Cast immobilization duration for distal radius fractures, a systematic review
Source: Eur J Trauma Emerg Surg. 2024 Mar 20;50(4):1621–36. doi: 10.1007/s00068-024-02494-y (PMC11458645; doi:10.1007/s00068-024-02494-y)
Supplement: Supplementary file 1 — Supplementary file1 (DOCX 20 KB) [file 68_2024_2494_MOESM1_ESM.docx]

Supplementary table 1

Search performed 24^th^ of January 2023

**Pubmed**

| **Search** | **Query** | **Records** |
| --- | --- | --- |
| **#3** | #1 AND #2 | 3.515 |
| **#2** | (“Conservative Treatment”[Mesh] OR conservativ*[tiab] OR non-operat*[tiab] OR non-surg*[tiab] OR nonoperat*[tiab] OR nonsurg*[tiab] OR “Casts, Surgical”[Mesh] OR “Splints”[Mesh] OR cast[tiab] OR casts[tiab] OR casting[tiab] OR plaster*[tiab] OR splint[tiab] OR splints[tiab] OR splinting[tiab] OR immobil*[tiab]) | 413.619 |
| **#1** | (“Radius Fractures”[Mesh] OR “Wrist Fractures”[Mesh] OR ((“Radius”[Mesh] OR radius[tiab]) AND fracture*[tiab]) OR “radial fracture”[tiab:~3] OR “colles fracture”[tiab:~3] OR “barton fracture”[tiab:~3] OR “barton’s fracture”[tiab:~3] OR “smith fracture”[tiab:~3] OR “smith’s fracture”[tiab:~3] OR “colles’s fracture”[tiab:~3] OR “radial fractures”[tiab:~3] OR “colles fractures”[tiab:~3] OR “barton fractures”[tiab:~3] OR “barton’s fractures”[tiab:~3] OR “smith fractures”[tiab:~3] OR “smith’s fractures”[tiab:~3] OR “colles’s fractures”[tiab:~3] OR “wrist fracture”[tiab:~3] OR “wrist fractures”[tiab:~3] OR "forearm fracture"[tiab:~3] OR "forearm fractures"[tiab:~3]) | 20.357 |

**Embase**

| **Search** | **Query** | **Records** |
| --- | --- | --- |
| **#3** | **#1 AND #2** | 4.333 |
| **#2** | (Conservative Treatment/ OR conservativ* .ti,ab,kf. OR non-operat* .ti,ab,kf. OR non-surg* .ti,ab,kf. OR nonoperat* .ti,ab,kf. OR nonsurg* .ti,ab,kf. OR orthopedic cast/ or arm cast/ or cast brace/ or fiberglass cast/ or exp plaster cast/ or soft cast/ or waterproof cast/ OR splint/ or exp arm splint/ or exp external splint/ or exp wrist splint/ OR cast .ti,ab,kf. OR casts .ti,ab,kf. OR casting .ti,ab,kf. OR plaster* .ti,ab,kf. OR splint .ti,ab,kf. OR splints .ti,ab,kf. OR splinting .ti,ab,kf. OR immobil* .ti,ab,kf.) | 546.417 |
| **#1** | (distal radius fracture/ or radius fracture/ or wrist fracture/ or barton fracture/ or colles fracture/ or smith fracture/ or ((radius/ or distal radius/) and fracture*.ti,ab,kf.) or ((radius or radial or colles or barton or barton's or smith or smith's or colles's or forearm or wrist) adj3 fracture*).ti,ab,kf.) | 23.267 |

**CINAHL**

| **Search** | **Query** | **Records** |
| --- | --- | --- |
| **#3** | **#1 AND #2** | 879 |
| **#2** | ((MH "Conservative Treatment") OR (MH "Casts") OR (MH "Splints") OR TI(conservativ* OR non-operat* OR non-surg* OR nonoperat* OR nonsurg* OR cast OR casts OR casting OR plaster* OR splint OR splints OR splinting OR immobil*) OR AB(cast OR casts OR casting OR plaster* OR splint OR splints OR splinting OR immobil* )) | 32.362 |
| **#1** | ((MH "Radius Fractures+") OR (MH "Wrist Fractures") OR ((MH "Radius") AND fracture*) OR TI ( (radius or radial or colles or barton or barton's or smith or smith's or colles's or forearm or wrist) N3 fracture* ) OR AB ( (radius or radial or colles or barton or barton's or smith or smith's or colles's or forearm or wrist) N3 fracture*)) | 5.366 |

**Cochrane Library**

| **Search** | **Query** | **Records** |
| --- | --- | --- |
| **#3** | #1 AND #2 | 823 |
| **#2** | ([mh "Conservative Treatment"] OR conservativ*:ti,ab,kw OR non-operat*:ti,ab,kw OR non-surg*:ti,ab,kw OR nonoperat*:ti,ab,kw OR nonsurg*:ti,ab,kw OR [mh "Casts, Surgical"] OR [mh Splints] OR cast:ti,ab,kw OR casts:ti,ab,kw OR casting:ti,ab,kw OR plaster*:ti,ab,kw OR splint:ti,ab,kw OR splints:ti,ab,kw OR splinting:ti,ab,kw OR immobil*:ti,ab,kw) | 27.492 |
| **#1** | ([mh "Radius Fractures"] OR [mh "Wrist Fractures"] OR (([mh Radius] OR radius:ti,ab,kw) AND fracture*:ti,ab,kw) OR (radial NEAR/3 fracture*):ti,ab,kw OR (colles NEAR/3 fracture*):ti,ab,kw OR (barton NEAR/3 fracture*):ti,ab,kw OR (barton’s NEAR/3 fracture*):ti,ab,kw OR (smith NEAR/3 fracture*):ti,ab,kw OR (smith’s NEAR/3 fracture*):ti,ab,kw OR (colles’s NEAR/3 fracture*):ti,ab,kw OR (wrist NEAR/3 fracture*):ti,ab,kw OR (forearm NEAR/3 fracture*):ti,ab,kw) | 2.578 |

**Web of Science**

| **Search** | **Query** | **Records** |
| --- | --- | --- |
| **#3** | **#1 AND #2** | 2.428 |
| **#2** | TS=(conservativ* OR non-operat* OR non-surg* OR nonoperat* OR nonsurg* OR cast OR casts OR casting OR plaster* OR splint OR splints OR splinting OR immobil*)) | 798.731 |
| **#1** | TS=(((radial NEAR/3 fracture*) OR (radius NEAR/3 fracture*) OR (colles NEAR/3 fracture*) OR (barton NEAR/3 fracture*) OR (barton’s NEAR/3 fracture*) OR (smith NEAR/3 fracture*) OR (smith’s NEAR/3 fracture*) OR (colles’s NEAR/3 fracture*) OR (wrist NEAR/3 fracture*) OR (forearm NEAR/3 fracture*)) | 14.055 |
